# Cardiovascular Events and Heart Failure in Patients With Type 2 Diabetes Treated With Dipeptidyl Peptidase-4 Inhibitors: A Meta-Analysis

**DOI:** 10.1016/j.curtheres.2025.100804

**Published:** 2025-07-15

**Authors:** Adili Tuersun, Shufen Cui, Li Han, Alimu Aikebaier, Yanyan Shi, Gang Cheng, Lei Cheng, Guo Ma

**Affiliations:** 1 School of Pharmaceutical Sciences, State Key Laboratory of Advanced Drug Formulations for Overcoming Delivery Barriers, Fudan University, Shanghai 201203, PR China; 2 Department of Pharmacy, Shandong University of Traditional Chinese Medicine Affiliated Hospital, Jinan, China; 3 Department of Pharmacy, The First People’s Hospital of Kashi Prefecture, Xinjiang, China; 4 Department of Pharmacy, Shenyang Pharmaceutical University, Shenyang, China

**Keywords:** cardiovascular death, cardiovascular outcomes, dipeptidyl peptidase 4 inhibitors, heart failure, type 2 diabetes mellitus

## Abstract

**Background:**

The global prevalence of type 2 diabetes mellitus (T2DM) continues to rise, with patients facing significantly elevated risks of cardiovascular complications, particularly heart failure (HF).

**Objective:**

This examination sought to methodically examine cardiovascular sequelae, notably heart failure, among users of dipeptidyl peptidase 4 (DPP-4) inhibitors when compared with nonusers.

**Methods:**

Cochrane, Embase, and PubMed databases, which compared the use of DPP-4 inhibitors and reported cardiovascular outcomes and heart failure events in patients with type 2 diabetes mellitus (T2DM), were searched using specific terms. Studies were included if they satisfied the following inclusion criteria: they were randomized trials comparing DPP-4 inhibitor use in patients with T2DM; study duration was longer than 24 weeks; and they reported cardiovascular outcomes as their main or secondary end points. Stata 15 MP (StataCorp LLC, College Station, Texas, USA) was used to analyze the data, and odds ratios (ORs) with 95% CIs were used to represent the results.

**Results:**

A total of 79,010 participants with T2DM were included. A total of 37,895 patients were assigned to the DPP-4 inhibitor group, whereas 41,115 patients were assigned to the control group. Results of the analysis showed that during a mean follow-up period ranging from 24 to 302 weeks, heart failure incidence did not differ significantly between T2DM patients treated with DPP-4 inhibitors and those who were not (OR = 1.06; 95% CI, 0.96–1.18; *P* = 0.452). Major adverse cardiovascular events (including nonfatal myocardial infarction, nonfatal stroke, and cardiovascular death; OR = 1.01; 95% CI, 0.95–1.08; *P* = 0.354), stroke (OR = 1.01; 95% CI, 0.78–1.30; *P* = 0.968), myocardial infarction (OR = 0.89; 95% CI, 0.73–1.07; *P* = 0.49), and all-cause mortality (OR = 1.03; 95% CI, 0.96–1.11; *P* = 0.309) were also similarly manifested in both groups.

**Conclusions:**

The present analysis showed that treatment with DPP-4 inhibitors did not significantly increase cardiovascular outcomes and heart failure in these patients with T2DM, indicating that these drugs may be safe to use in terms of cardiovascular events.

## Graphical abstract

**Figure:**
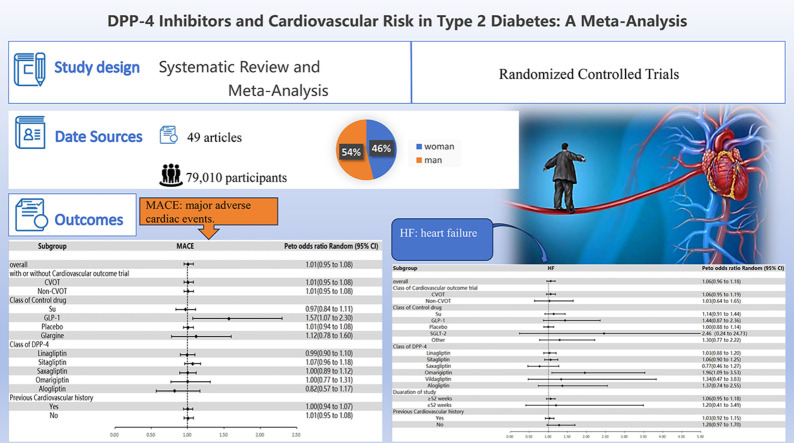

## Introduction

Approximately 537 million adults worldwide have diabetes, most of whom have type 2 diabetes mellitus (T2DM), and this number is expected to increase to 783 million by 2045.^1^^,^^2^ The prevalence of heart failure (HF) is approximately 2.5-fold higher in individuals with diabetes than in the general population, and an approximate of 49% of patients with HF are postulated to concurrently endure diabetes. Additionally, patients comorbidly experiencing both T2DM and HF experience exacerbated clinical manifestations when compared with patients with HF alone but no concurrent diabetes.^3^^,^^4^

Since 2008, the US Food and Drug Administration mandated the demonstration of cardiovascular safety as a crucial prerequisite for the approval of novel glucose-regulating drugs,^5^ precipitating dedicated cardiovascular outcome trials. Data derived from randomized controlled trials (RCTs) have unequivocally demonstrated that dipeptidyl peptidase 4 (DPP-4) inhibitors mitigate levels of glycated hemoglobin (HbA_1c_),^6^^,^^7^ do not impact body weight,^6^ present a diminished risk of hypoglycemia,^8^^,^^9^ and do not escalate the likelihood of cardiovascular incidents.^10^, ^11^, ^12^, ^13^

A pivotal trial^14^ (SAVOR-TIMI 53 (Saxagliptin Assessment of Vascular Outcomes Recorded in Patients with Diabetes Mellitus–Thrombolysis in Myocardial Infarction 53)) showed an amplified risk of HF (hazard ratio [HR] = 1.27; 95% CI, 1.07–1.51) associated with the DPP-4 inhibitor saxagliptin. Although unexpected, this observation sparked concern among medical professionals and health regulatory entities. Subsequently, the EXAMINE (Examination of Cardiovascular Outcomes with Alogliptin versus Standard of Care) trial,^15^ which evaluated alogliptin efficacy, and the TECOS (Trial Evaluating Cardiovascular Outcomes with Sitagliptin) trial,^16^ which scrutinized sitagliptin’s value, concluded no substantial influence on hospitalization for HF. Reports derived from observational investigations have shown inconsistency,^17^, ^18^, ^19^, ^20^ casting doubt upon the impact of DPP-4 inhibitors on HF.

Existing meta-analyses and observational studies on the association between DPP-4 inhibitors and HF risk have reported heterogeneous findings. While the SAVOR-TIMI 53 trial highlighted a 27% increased risk of HF hospitalization with saxagliptin (HR = 1.27; 95% CI, 1.07–1.51), other DPP-4 inhibitors such as sitagliptin (HR = 1.00; 95% CI, 0.83–1.20) and linagliptin (HR = 0.90; 95% CI, 0.74–1.08) demonstrated neutral effects in similar trials.^14^ A 2021 meta-analysis of 182 RCTs further confirmed the overall cardiovascular safety of DPP-4 inhibitors as a class, showing no significant association with major adverse cardiovascular events (MACEs), all-cause mortality, or HF risk (HR = 1.05; 95% CI, 0.96–1.15). However, subgroup analyses revealed saxagliptin as an exception, with a 22% increased HF risk (HR = 1.22; 95% CI, 1.03–1.45).^21^ Conversely, a large observational cohort study comparing DPP-4 inhibitors with glucagon-like peptide 1 (GLP-1) receptor agonists (RAs) reported a 14% reduced risk of HF hospitalization among DPP-4 inhibitor users (HR = 0.86; 95% CI, 0.83–0.90), particularly for saxagliptin (HR = 0.74) and sitagliptin (HR = 0.92).^22^ These conflicting findings underscore the need to clarify the cardiovascular profile of individual DPP-4 inhibitors and contextualize their risks relative to other antihyperglycemic drugs.

Building on prior meta-analyses,^21^^,^^23^ the present study extends the evidence base by incorporating 21 recent RCTs (RCTs^24^, ^25^, ^26^, ^27^, ^28^ that were not included in previous studies) and conducting prespecified subgroup analyses. This approach allows a more nuanced assessment of cardiovascular safety across diverse patient populations, addressing gaps in previous syntheses that focused on pooled class effects without stratification.

## Methods

This systematic review is reported according to the Preferred Reporting Items for Systematic Reviews and Meta-Analyses guidelines^29^ and was conducted according to a registered protocol (International Prospective Register of Systematic Reviews (PROSPERO) identifier: CRD42023458356).

### Eligibility criteria

We incorporated RCTs that contrastively examined DPP-4 inhibitors versus placebo or active glucose-lowering medications among adult patients afflicted with T2DM, necessitating sustained follow-up for a minimum duration of 24 weeks. We cataloged study methodologies on the basis of the protocol established by the Cochrane Non-Randomized Studies Methods Group. Trials, principally Phase III studies, acknowledged HF either as a routine adverse reaction or significant adverse event. For critical adverse events, hospitalization due to HF might have been noted.

### Literature search

We performed a systematic search of scientific literature in the following databases (from inception through November 1, 2024): PubMed, Embase, and Cochrane. Details of the search terms are provided in **Supplemental Table 1**). We also searched the ClinicalTrials.gov website to collect data from completed but unpublished trials, and we manually searched the reference lists of potentially relevant studies for any additional studies. Systematic reviews were identified and hand-screened for additional trials. Two investigators (A.T. and L.H.) developed selection criteria and screened the titles and abstracts of search results for relevance after the removal of duplicates. The full texts of the remaining results were independently assessed by 2 authors (A.T. and L.H.) for inclusion on the basis of predetermined criteria. Discrepancies between reviewers were resolved by consensus or adjudicated by a third reviewer.

### Risk of bias assessment

We used the Cochrane Collaboration’s instrument to appraise the risk of bias in RCTs. This instrument encompassed the dimensions of random sequence generation, allocation concealment, blinding of participants, caregivers, and outcome assessors (ie, HF or hospital admission for HF), as well as the adjudication of outcomes.

### Data collection

The principal end point was MACE, and the secondary end point was HF. We collected the following information from each eligible RCT: (1) General study characteristics: author name, year of publication, total number of patients randomized, number of treatment groups, length of follow-up, trial registry number; (2) patient characteristics: sex, age, diabetes duration, body mass index, and baseline HbA_1c_ level; (3) interventions: details of DPP-4 inhibitor treatments and control group (eg, drug generic name); and (4) Outcomes: the definition of HF, number of events, and patients included for analyses in each group, MACEs, all-cause mortality, myocardial infarction (MI), and stroke (hemorrhagic stroke or ischemic stroke) in each arm; when they were not listed as adverse events of special interest, we collected only cases reported as serious adverse events.

For each trial, if the initial intervention assignment was altered (eg, patients within the placebo cohort commenced receiving DPP-4 inhibitors after 24 weeks), we procured the outcome data before that point. If a trial had multiple reports, we collated all data into one study. If an experiment incorporated reports from both ClinicalTrials.gov and published literature, we meticulously verified data from these 2 sources for congruence. If outcome statistics were communicated at multiple follow-up intervals, we employed data from the most extensive follow-up.

### Data analysis

Stata 15 MP software (StataCorp LLC, College Station, Texas, USA) was used to carry out statistical analysis of the pooled data. Peto odds ratios (ORs) and 95% CIs were generated to represent the main analytical data throughout the results section.

Expected heterogeneity was assessed using the (1) Q statistical analysis, whereby an outcome exhibiting a *P* value ≤0.05 was deemed statistically significant; and (2) the *I*^2^ statistical algorithm, whereby a smaller *I*^2^ score indicated diminished heterogeneity.

A random statistical effect model was applied in this analysis; we pooled outcome data using Peto’s methods because of very low event rates.^30^ We explored sources of heterogeneity with 5 prespecified subgroup hypotheses: (1) type of control drug (in subgroup analysis, other indicated sulfonylureas or thiazolidinediones); (2) length of follow-up (≤52 and >52 weeks; (3) individual DPP-4 inhibitors (different DPP-4 inhibitors vs control drug); (4) any previous cardiovascular events (trials not explicitly reporting this information were considered as not excluding patients with cardiovascular events); and (5) trials with cardiovascular or noncardiovascular outcomes.

## Results

### Trial characteristics

**Fig. 1:**
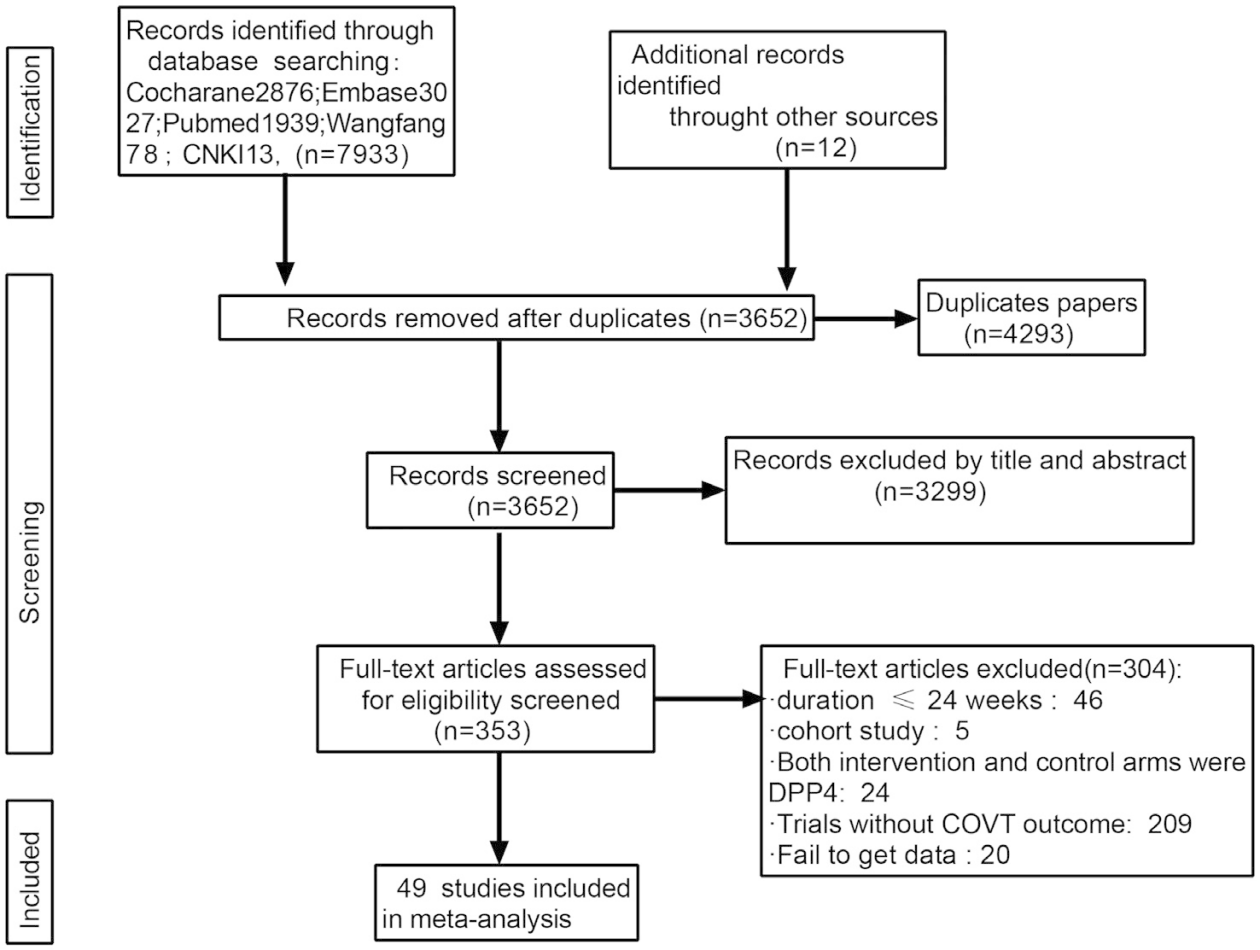
Flowchart of article selection. CVOT = cardiovascular outcome trial; CNKI, China National Knowledge Infrastructure; DPP4 = dipeptidyl peptidase-4.

In total, 7933 articles were identified in the primary search from databases, and 12 additional studies were identified through other sources (published meta-analyses and reviews). Among them, in addition to 4293 duplicates, there were 3299 unrelated studies, and 304 studies were discarded for the following reasons: duration <24 weeks, non-RCT designs, both treatment and control groups received DPP-4 inhibitors, or trials that did not report any results related to this study. Finally, 49 articles met the inclusion criteria.^14^, ^15^, ^16^^,^^24^, ^25^, ^26^, ^27^, ^28^^,^^31^, ^32^, ^33^, ^34^, ^35^, ^36^, ^37^, ^38^, ^39^, ^40^, ^41^, ^42^, ^43^, ^44^, ^45^, ^46^, ^47^, ^48^, ^49^, ^50^, ^51^, ^52^, ^53^, ^54^, ^55^, ^56^, ^57^, ^58^, ^59^, ^60^, ^61^, ^62^, ^63^, ^64^, ^65^, ^66^, ^67^, ^68^, ^69^, ^70^, ^71^ The flowchart of study selection is presented in Figure 1.

The features of the 49 trials incorporated into the assessment are delineated in **Supplemental Table 2**. The general caliber was satisfactory in most trials across all items of the Cochrane instrument, except for “Blindness of participants and personnel,” which cannot be outrightly dismissed for several trials (open-label design or methodologies inadequately characterized; **Supplemental Figure 1**).

### MACEs

Within 49 studies presenting data (37,895 patients in the DPP-4 inhibitors group and 41,115 patients in the control cohort), 11 studies reported at least 1 occurrence (2404 and 2366 events among individuals receiving DPP-4 inhibitors and comparator drugs, respectively). Evidence of publication bias was not detected upon visual analysis of the funnel plot (**Supplemental Figure 2**), as endorsed by Egger’s test (*P* = 0.960).

**Fig. 2:**
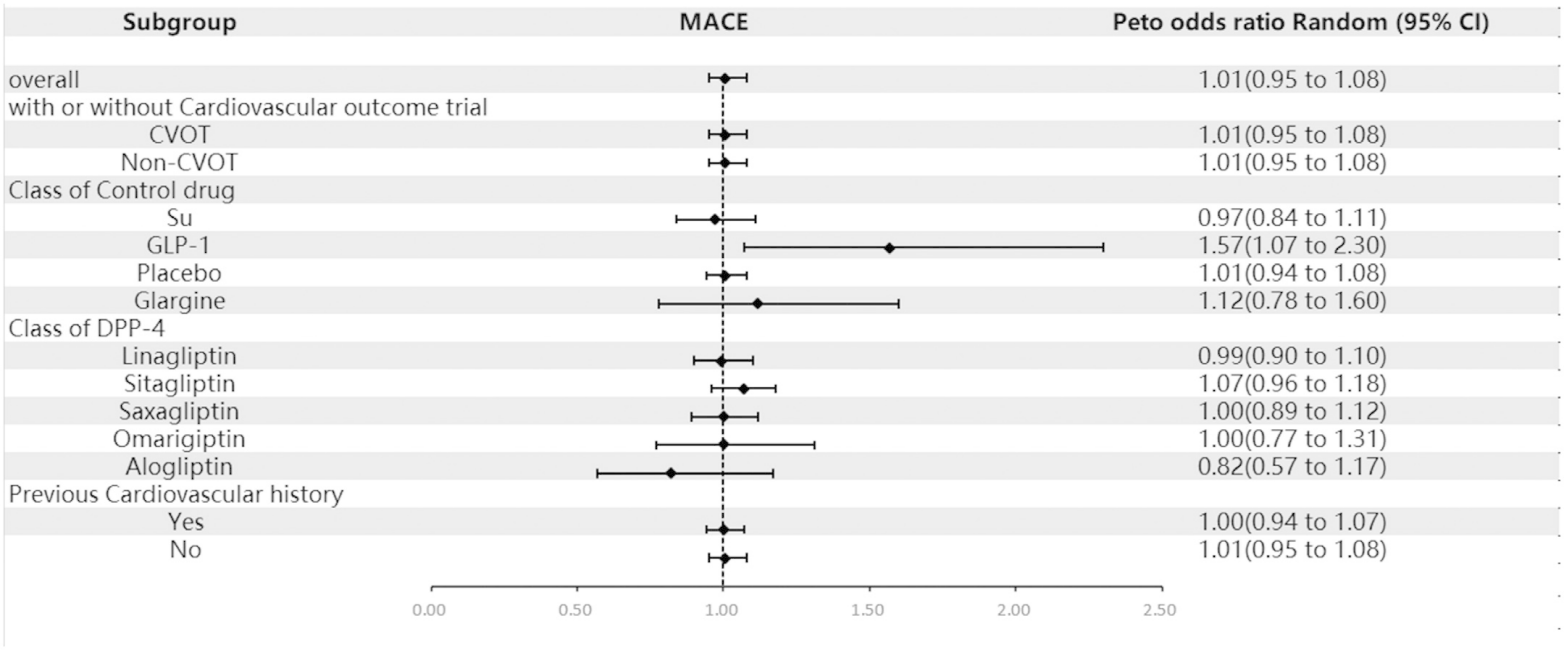
Overall and subgroup results of included studies for major adverse cardiovascular events (MACEs). CVOT = cardiovascular outcome trial; DPP-4 = dipeptidyl peptidase-4; GLP-1 = glucagon-like peptide-1; non-CVOT = trial without cardiovascular outcome; Su = sulfonylurea.

Dipeptidyl peptidase 4 inhibitors showed no significant increase in susceptibility toward MACEs (OR = 1.01; 95% CI, 0.95–1.08; Figure 2), with no evidence of heterogeneity (*I*^2^ = 9.2%). Subgroup analyses showed that when analyzed by RCT with or without cardiovascular end points, the OR for the group with cardiovascular end points included was 1.01 (95% CI, 0.95–1.08), and the OR for the group without cardiovascular end points included was 1.01 95% CI, 0.95–1.08). When analyzed by the type of drug in the control group, the OR for DPP-4 inhibitors to sulfonylureas was 0.97 (95% CI, 0.84–1.11), for GLP-1 RAs was 1.57 (95% CI, 1.07–2.30), for the placebo was 1.01 (95% CI, 0.94–1.08), and for glargine was 1.12 (95% CI, 0.78–1.60). When analyzed by subgroups of DPP-4 inhibitors, the OR for linagliptin was 0.99 (95% CI, 0.90–1.10), for sitagliptin was 1.07 (95% CI, 0.96–1.18), for saxagliptin was 1.00 (95% CI, 0.89–1.12), for omarigliptin was 1.00 (95% CI, 0.77–1.31), and for alogliptin was 0.82 (95% CI, 0.57–1.17). When analyzed by previous history of cardiovascular disease, the OR for the group with a history of cardiovascular disease was 1.00 (95% CI, 0.94–1.07) and for the group without a history of cardiovascular disease was 1.01 (95% CI, 0.95–1.08; Figure 2 and **Supplemental Figure 3**). The results showed that DPP-4 inhibitors did not significantly increase the risk of MACEs.

### All-cause death

An investigation involving 29 studies reported at least 1 event (1574 and 1185 with DPP-4 inhibitors and comparators, respectively). Evidence of publication bias was not detected upon visual analysis of the funnel plot (**Supplemental Figure 4**), as Egger’s test *P* value was 0.402.

**Fig. 3:**
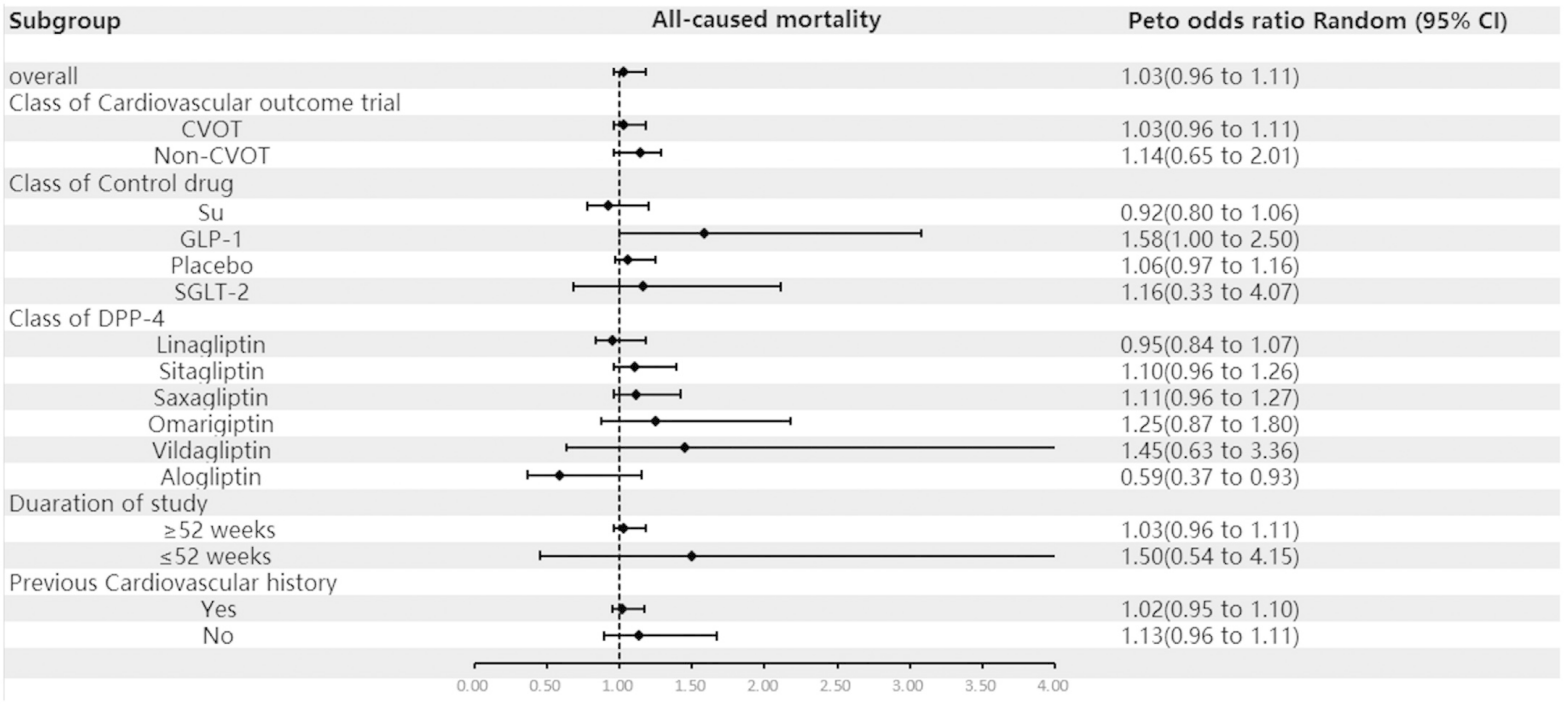
Overall and subgroup results of included studies for all-cause mortality. CVOT = cardiovascular outcome trial; DPP-4 = dipeptidyl peptidase-4; GLP-1 = glucagon-like peptide-1; non-CVOT = trial without cardiovascular outcome; SGLT-2 = sodium-glucose cotransporter-2; Su = sulfonylurea.

The influence of DPP-4 inhibitors on MACE did not exhibit a significant difference between control groups (OR = 1.03; 95% CI, 0.96–1.11; Figure 3), with no indication of heterogeneity (*I*^2^ = 9.6%). Subgroup evaluations indicated that, when scrutinized by RCTs with or without cardiovascular end points, the OR for the group including cardiovascular end points was 1.03 (95% CI, 0.96–1.11), and the OR for the group without cardiovascular end points was 1.14 (95% CI, 0.65–2.01). When classified by the type of medication used as the control group, the OR for DPP-4 inhibitors to sulfonylureas was 0.92 (95% CI, 0.80–1.06), for GLP-1 was 1.58 (95% CI, 1.00–2.50), for placebo was 1.06 (95% CI, 0.97–1.16), and for sodium-glucose cotransporter-2 (SGLT-2) was 1.16 (95% CI, 0.33–4.07). When segmented by subcategories of DPP-4 inhibitor type, the OR for linagliptin was 0.95 (95% CI, 0.84–1.07), for sitagliptin was 1.10 (95% CI, 0.96–1.26), for saxagliptin was 1.11 (95% CI, 0.96–1.27), for omarigliptin was 1.25 (95% CI, 0.87–1.80), for vildagliptin was 1.45 (95% CI, 0.63–3.36), and for alogliptin was 0.59 (95% CI, 0.37–0.93). When segmented by prior history of cardiovascular disease, the OR for the group having such a history was 1.02 (95% CI, 0.95–1.10) and for the group without such a history was 1.13 (95% CI, 0.96–1.11); when examined by subsets of duration of follow-up weeks, the OR for >52 weeks was 1.03 (95% CI, 0.96–1.11) and that for <52 weeks was 1.50 (95% CI, 0.54–4.15; Figure 3 and **Supplemental Figure 5**). The results showed that DPP-4 inhibitors did not significantly increase the risk of all-cause mortality.

### HF

Many RCTs indicated that HF occurring after randomization was adjudicated by a clinical end point committee. Some included trials that defined HF events requiring either (1) Hospitalization for worsening HF with ≥24-hour stay accompanied by: new/worsening symptoms (dyspnea and orthopnea) AND objective evidence of pulmonary congestion (chest X-ray) or reduced cardiac function (left ventricular ejection fraction <40% on echocardiography) (TECOS and SAVOR-TIMI 53 trials) OR (2) Emergency department visits with intravenous diuretic/nitrate administration AND elevated N-terminal pro-B-type natriuretic peptide >900 pg/mL (CARMELINA (Cardiovascular and Renal Microvascular Outcome Study With Linagliptin) and EXAMINE trials).

An investigation involving 41 studies reported at least 1 event (621 and 598 with DPP-4 inhibitors and comparators, respectively). Evidence of publication bias was not discernable via visual examination of the funnel plot (**Supplemental Figure 6**), as endorsed by Egger’s test (*P* = 0.403).

**Fig. 4:**
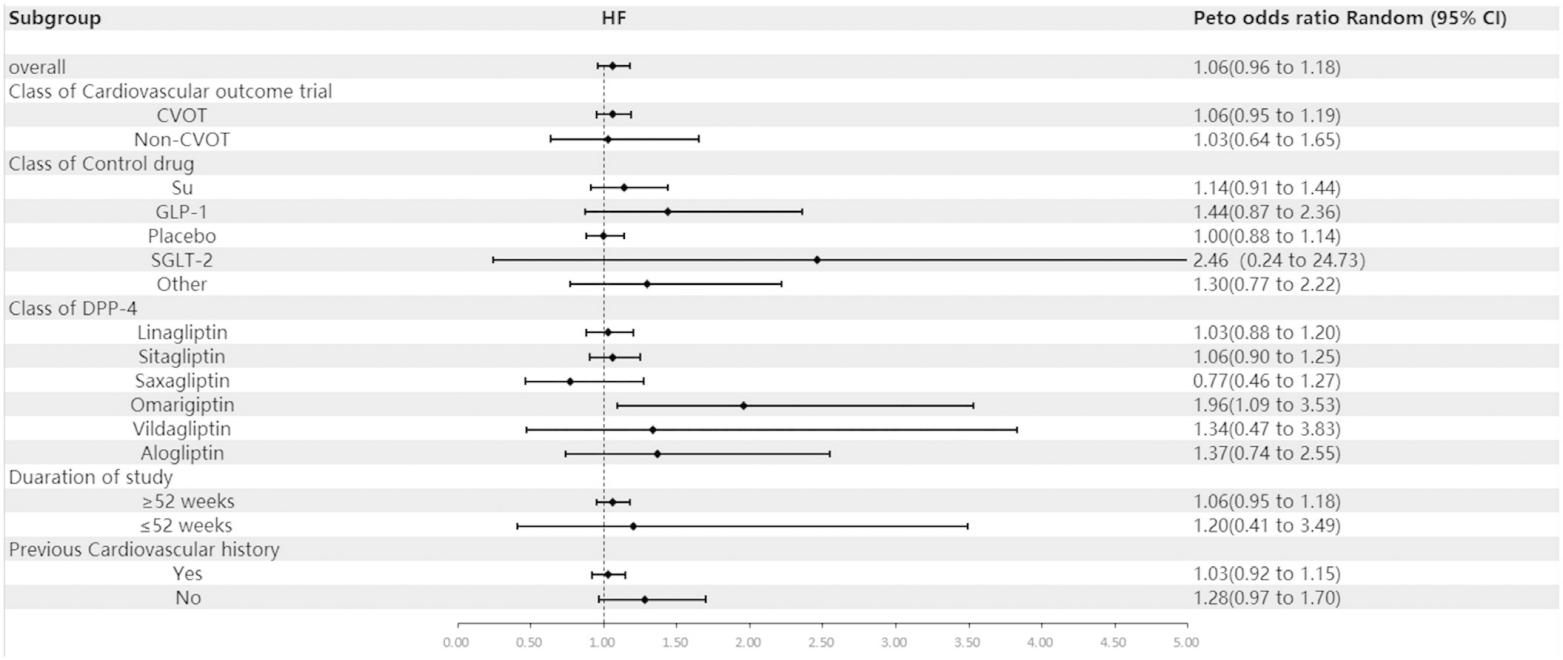
Overall and subgroup results of included studies for heart failure (HF). CVOT = cardiovascular outcome trial; DPP-4 = dipeptidyl peptidase-4; GLP-1 = glucagon-like peptide-1; non-CVOT = trial without cardiovascular outcome; SGLT-2 = sodium-glucose cotransporter-2; Su = sulfonylurea.

The engagement of DPP-4 inhibitors in HF demonstrated an insignificant disparity in the control group (OR = 1.06; 95% CI, 0.96–1.18; Figure 4), lacking evidence of heterogeneity (*I*^2^ = 1%). Exploratory subgroup evaluations revealed that when stratified according to whether RCTs were designed with or without cardiovascular endpoints, the OR associated with the group incorporating cardiovascular end points equaled 1.06 (95% CI, 0.95–1.19), whereas the OR corresponding to the group without cardiovascular end points was 1.03 (95% CI, 0.64–1.65). Qualifying according to the class of medication used as the control arm, the OR related to DPP-4 inhibitors in comparison with sulfonylureas was 1.14 (95% CI, 0.91–1.14), for the GLP-1 regime was 1.44 (95% CI, 0.87–2.36), for the placebo treatment was 1.00 (95% CI, 0.88–1.14), and for SGLT-2 usage was 2.46 (95% CI, 0.24–24.73). Segmentation based on subcategories of DPP-4 inhibitors revealed the OR for linagliptin was 1.03 (95% CI, 0.88–1.20), for sitagliptin was 1.06 (95% CI, 0.90–1.25), for saxagliptin was 0.77 (95% CI, 0.46–1.27), for omarigliptin was 1.96 (95% CI, 1.09–3.53), for vildagliptin was 1.34 (95% CI, 0.47–3.83), and for alogliptin was 1.37 (95% CI, 0.74–2.55). After further categorizing on the basis of previous history of cardiovascular ailment, the OR for the group possessing this attribute was 1.03 (95% CI, 0.92–1.15) versus 1.28 (95% CI, 0.97–1.70) for those lacking such a history. Upon examining cohorts segmented by duration of follow-up weeks, the OR for >52 weeks was 1.06 (95% CI, 0.95–1.18), whereas that for <52 weeks, it was 1.20 (95% CI, 0.41–3.49; Figure 4 and **Supplemental Figure 7**). This finding corroborates that DPP-4 inhibitors did not significantly amplify the risk of HF.

### MI

An investigation involving 39 studies reported at least 1 event (201 and 173 with DPP-4 inhibitors and comparators, respectively). Evidence of publication bias was not discernable via visual examination of the funnel plot (**Supplemental Figure 8**), as endorsed by Egger’s test (*P* = 0.487).

**Fig. 5:**
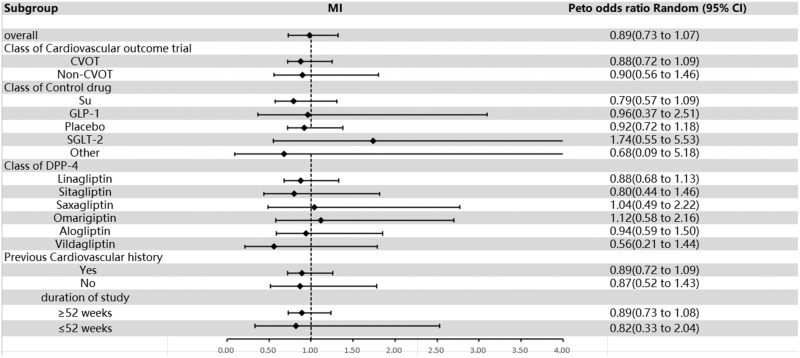
Overall and subgroup results of included studies for myocardial infarction (MI). CVOT = cardiovascular outcome trial; DPP-4 = dipeptidyl peptidase-4; GLP-1 = glucagon-like peptide-1; non-CVOT = trial without cardiovascular outcome; SGLT-2 = sodium-glucose cotransporter-2; Su = sulfonylurea.

Dipeptidyl peptidase 4 inhibitors did not significantly increase the risk of MI compared with controls (OR = 0.89; 95% CI, 0.73–1.07; Figure 5), and there was no heterogeneity (*I*^2^ = 0%). Subgroup analyses showed that when considering RCTs with or without cardiovascular end points, the group with cardiovascular end points had an OR of 0.88 (95% CI, 0.72–1.09), whereas the group without cardiovascular end points had an OR of 0.90 (95% CI, 0.56–1.46). When analyzed by control drug type, DPP-4 inhibitors had an OR of 0.79 (95% CI, 0.57–1.09) compared with sulfonylureas, 0.96 (95% CI, 0.37–2.51) compared with GLP-1 RAs, 0.92 (95% CI, 0.72–1.18) compared with the placebo group, 0.68 (95% CI, 0.09–5.18) compared with the other groups, and 1.74 (95% CI, 0.55–5.53) compared with SGLT-2. Subgrouping based on DPP-4 inhibitor type showed an OR of 0.88 (95% CI, 0.68–1.13) for linagliptin, 0.80 (95% CI, 0.44–1. 46) for sitagliptin, 1.04 (95% CI, 0.49–2.22) for saxagliptin, 1.12 (95% CI, 0.58–2.16) for omarigliptin, 0.56 (95% CI, 0.21–1.44) for vildagliptin, and 0.94 for alogliptin (95% CI, 0.59–1.50). Further analysis according to previous cardiovascular history showed an OR of 0.89 (95% CI, 0.72–1.09) for those with a history of cardiovascular disease and an OR of 0.87 (95% CI, 0.52–.43) for those without a history of cardiovascular disease. When divided by weeks of follow-up, the OR for those with >52 weeks of follow-up was 0.89 (95% CI, 0.72–1.08), whereas the OR for those with <52 weeks of follow-up was 0.82 (95% CI, 0.33–2.04; Figure 5 and **Supplemental Figure 9**). These results suggest that DPP-4 inhibitors did not significantly increase the risk of MI.

### Stroke

**Fig. 6:**
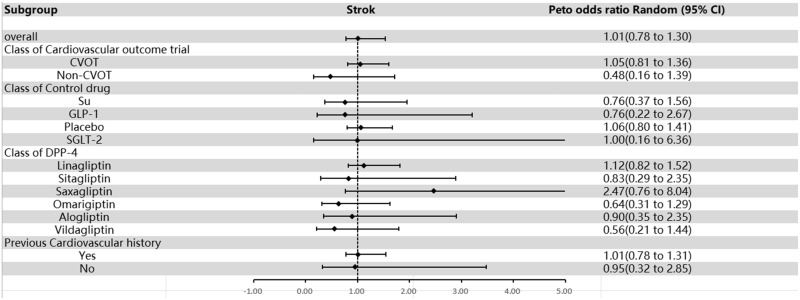
Overall and subgroup results of included studies for stroke. CVOT = cardiovascular outcome trial; DPP-4 = dipeptidyl peptidase-4; GLP-1 = glucagon-like peptide-1; non-CVOT = trial without cardiovascular outcome; SGLT-2 = sodium-glucose cotransporter-2; Su = sulfonylurea.

An investigation involving 15 studies reported at least 1 event (121 and 122 with DPP-4 inhibitors and comparators, respectively). Evidence of publication bias was not discernable via visual examination of the Funnel plot (**Supplemental Figure 10**), as endorsed by Egger’s test (*P* = 0.670). Dipeptidyl peptidase 4 inhibitors did not significantly increase the risk of stroke compared with controls (OR= 1.01; 95% CI, 0.78–1.30; Figure 6), and there was no heterogeneity (*I*^2^ = 39.8%, *P* = 0.056). Subgroup analyses showed that when considering RCTs with or without cardiovascular end points, the group with cardiovascular end points had an OR of 1.05 (95% CI, 0.81–1.36), whereas the group without cardiovascular end points had an OR of 0.48 (95% CI, 0.16–1.39). When analyzed by control drug type, DPP-4 inhibitors had an OR of 0.76 (95% CI, 0.37–1.56) compared with sulfonylureas, 0.76 (95% CI, 0.22–2.67) compared with GLP-1, 1.06 (95% CI, 0.80–1.41) compared with the placebo group, and 1.00 (95% CI, 0.16–6.36) compared with SGLT-2. Subgrouping based on DPP-4 inhibitors showed an OR of 1.12 (95% CI, 0.82–1.52) for linagliptin, 0.83 (95% CI, 0.29–2.35) for sitagliptin, 2.47 (95% CI, 0.76–8.04) for saxagliptin, 0.64 (95% CI, 0.31–1.29) for omarigliptin, 0.56 (95% CI, 0.21–1.44) for vildagliptin, and 0.90 for alogliptin (95% CI, 0.35–2.35). Further analysis according to previous cardiovascular history showed an OR of 1.01 (95% CI, 0.78–1.31) for those with a history of cardiovascular disease and an OR of 0.95 (95% CI, 0.32–2.85) for those without a history of cardiovascular disease (Figure 6 and **Supplemental Figure 11**). This result suggests that DPP-4 inhibitors did not significantly increase the risk of stroke.

## Discussion

The findings reported in this article indicate that DPP-4 inhibitors have a neutral impact on all-cause mortality, major cardiovascular events, myocardial infarction, heart failure, and stroke. Our findings align with prior evidence on the safety of DPP-4 inhibitors in diabetic populations. For instance, a previous meta-analysis by our group^72^ demonstrated no increased risk of adverse renal events with DPP-4 inhibitor use, further supporting their overall safety profile. While the present study focuses on cardiovascular outcomes, the consistency across organ systems underscores the potential class-wide safety of these agents. We assessed the risk of bias in the included RCTs using the Cochrane Collaboration’s instrument, which covers key aspects such as random sequence generation, allocation concealment, and blinding of participants, caregivers, and outcome assessors. The results are shown in Supplemental Figure 1 risk of bias graph and risk of bias summary. Overall, the included studies exhibited a low risk of bias, indicating the reliability of our findings.

The implementation of DPP-4 inhibitors has demonstrated promising effects on cardiac risk markers^73^^,^^74^ as well as vascular performance.^75^ This has raised significant research interest in their potential to mitigate cardiovascular complications. Nevertheless, a multitude of cardiac outcome trials and comprehensive meta-analyses failed to corroborate these presumptions.^76^, ^77^, ^78^, ^79^, ^80^, ^81^ This meta-analysis stands out for its inclusion of substantial and latest RCTs that corroborate the cardiovascular tolerability of this medication class, demonstrating no elevation or diminishment in the occurrence of MACEs, aligning with prior meta-analyses.^76^, ^77^, ^78^, ^79^, ^80^, ^81^ Concurrently, the findings of this study regarding the influence of DPP-4 inhibitors on overall mortality exemplify neutrality, echoing past reports.^20^^,^^77^ Overall, we found that DPP-4 inhibitors did not significantly reduce all-cause mortality, HF, MACEs, MI, or stroke when compared with the control group over the 24- to 302-week study period. We performed subgroup analyses by study period, previous history of cardiovascular disease, presence of cardiovascular end points, type of control drug, and type of DPP-4 drug, which all remained uniform with the primary outcome, with no further new findings.

However, some meta-analyses have shown a reduction in HF,^22^ MACEs,^10^^,^^82^, ^83^, ^84^ and all-cause mortality^10^^,^^82^ with DPP-4 inhibitors. Several of these comprehensive evaluations were conducted exclusively on trials evaluating DPP-4 inhibitors versus sulfonylureas.^82^, ^83^, ^84^ The latter class of agents has been suggested to potentially increase the occurrence of a MACE or its elements (such as nonfatal stroke^85^^,^^86^) based on multiple research studies.^10^^,^^86^, ^87^, ^88^ Consequently, these outcomes likely reflect the inferior cardiovascular safety profile of sulfonylureas rather than any cardioprotective benefit of DPP-4 inhibitors.

The effect of DPP-4 inhibitors on HF is a controversial issue. The pivotal SAVOR-TIMI 53 study^14^ offered compelling evidence on cardiac safety and performance enhancements achieved by DPP-4 inhibitors. Nonetheless, no augmentation in cardiovascular incidents was detected during their administration. Of note, however, there was a notable surge in hospitalizations related to HF. The EXAMINE study,^15^ which involved a randomized sample of 1398 participants, demonstrated that patients treated with alogliptin exhibited significantly lower mortality rates within the specified subgroup. The CARMELINA trial,^28^ whose objective was to affirm the effects of linagliptin on cardiovascular and renal end points, revealed that the addition of linagliptin to standard therapy demonstrated noninferiority to placebo with regard to the primary end point of 3-point MACEs and failed to provide compelling evidence of cardiovascular therapeutic efficacy. Our analysis revealed a numerically elevated—although statistically marginal—MACE risk with DPP-4 inhibitors versus GLP-1 RAs (OR = 1.57; 95% CI, 1.07–2.30). This comparison was derived exclusively from the GRADE (Glycemia Reduction Approaches in Diabetes: A Comparative Effectiveness Study) trial^24^ (N = 2537), in which sitagliptin demonstrated higher event rates (67 of 1268, 5.28%) than liraglutide (43 of 1269, 3.39%). The observed trend may reflect: (1) confounding by pleiotropic effects (eg, GLP-1 RA–associated weight reduction and blood pressure lowering), and (2) limited power (contributing only 2.42% weight to the overall analysis). Similarly, the implausibly wide HF risk estimate for DPP-4 inhibitors versus SGLT-2 inhibitors (OR = 2.46; 95% CI, 0.24–24.73) stems from extreme data sparsity: only 3 trials^43^^,^^56^^,^^69^ (N = 1016) reported 3 total HF events (DPP-4 inhibitors: 2 vs SGLT-2 inhibitors: 1). Our analysis lacked precision to robustly compare drug classes due to insufficient head-to-head RCTs. These subgroup findings should thus be interpreted as hypothesis-generating, underscoring the need for future trials directly comparing contemporary glucose-lowering agents in standardized populations. The latest randomized clinical trial^24^ evaluated the superior efficacy between 4 popularly prescribed glucose regulation drugs (glargine, glimepiride, liraglutide, and sitagliptin) when combined with metformin. It revealed that throughout an average duration of 5.0 years and a study population comprising 5047 participants, there was no significant difference observed among the treatment groups concerning MACEs, hospital admissions due to HF, mortality resulting from cardiac diseases, or overall mortality, which is in line with our reported data.

The specific mechanistic pathway through which DPP-4 inhibitors potentially mitigate the progression of HF remains elusive. A recently completed, single-blind randomized clinical trial revealed that DPP-4 inhibitors possessed no discernible clinical implications on B-type natriuretic peptide, a crucial HF biomarker. The investigation posited that modifications in the concentration of HF biomarkers over time are predominantly associated with the inherent trajectory of HF rather than directly attributable to the impact of DPP-4 inhibitors.^89^ Nonetheless, a multitude of alternative mechanisms may underline the influence of DPP-4 inhibitors on HF. A significant impact was demonstrated in animal experiments where the constant stimulation of gastric inhibitory peptide resulted in hindering the development of atherosclerotic lesions and restraining the invasion of macrophages into the aortic wall.^90^^,^^91^ Evidence indicates that DPP-4 inhibitors are linked with enhancing left ventricular and endothelial operations, thereby postponing the onset or exacerbation of HF, primarily owing to its influence on blood pressure regulation, vascular function, and cardiac myocyte performance.^92^^,^^93^ Thus, the pooled estimate of the effect remained consistent with the latest RCTs.

As shown in Figure 3, DPP-4 inhibitor treatment seems to be associated with a nonsignificant but substantial increase in the risk of all-cause mortality compared with GLP-1. The significant decline in HbA_1c_ and fasting glucose values, along with a notable decrease in body weight observed through the application of GLP-1 agonists, supersedes those seen with DPP-4 inhibitors.^94^^,^^95^ The UK Prospective Diabetes Study demonstrated the beneficial effect of extended, rigorous glucose regulation on reducing mortality,^96^ which may explain the result we found in the subgroup analysis.

Regarding MI, our evaluation established that DPP-4 inhibitors did not significantly increase the risk of MI compared with either placebo or active medication. An earlier study suggested that saxagliptin might reduce MI risk compared with placebo.^97^ However, the small sample size and low event count render this finding likely a chance association, rather than a sustained effect, and may be subject to bias.

### Strengths and limitations

The literature search for this meta-analysis was conducted up to November 1, 2024. This ensures that our analysis is based on the most up-to-date information available at the time of the search. A considerable number of participants were involved, and preplanned analyses were conducted alongside double data abstraction verification and maintaining superior trial quality. This strategic approach significantly diminishes potential biases, most crucially reflected through the nonexistence of interstudy heterogeneity. Additionally, a multi-subgroup assessment was performed, adding further depth to the investigation.

Our study has limitations as well. First, HF classification varies significantly among clinical studies and displays inconsistencies and diversity within its boundaries. Second, in cardiovascular end point experiments, HF is generally recognized as a syndrome resulting in hospital admission, while noncardiac trials encompass all those incidentally reported as severe adverse incidents related to HF. Third, the present meta-analysis encompasses trials with varying durations, spanning between 24 and 304 weeks. It is conceivable that such variations in study length could potentially influence the findings related to the specific outcome variables under examination. Fourth, in the subgroup analysis comparing DPP-4 inhibitors with SGLT-2 inhibitors, we observed an implausibly wide CI for HF risk (OR = 2.46; 95% CI, 0.24–24.73). This is likely attributable to the extremely limited number of trials (n = 3) and sparse event data (only 3 total HF events reported), which precludes robust conclusions. Although this finding suggests a potential signal, the result should be interpreted with caution due to the lack of statistical power. Future studies directly comparing these drug classes in adequately powered trials are needed to clarify their relative effects on HF risk. Despite this, the statistical evaluations did not reveal evident heterogeneity among the trials examined, although it must be noted that the *I*^2^ test may underestimate the level of heterogeneity when there are a considerable number of studies included.

## Conclusion

Our investigation demonstrated that administration of DPP-4 inhibitors in individuals afflicted with T2DM does not notably elevate the cardiac risk within this demographic, inclusive of MI, MACE, HF, and all-cause mortality as well as stroke events. Presently, it may be conjectured that DPP-4 inhibitors could be deemed an efficacious medication for patients battling diabetes without considerable complications.

## Funding

This study was supported by the 10.13039/501100001809National Natural Science Foundation of China (grant numbers 82074109, 81873078, 81374051, and 81773687, 82374133).

## CRediT Authorship Contribution Statement

**Adili Tuersun:** Writing – original draft, Project administration, Investigation, Formal analysis, Data curation, Conceptualization. **Shufen Cui:** Writing – original draft, Project administration, Investigation, Formal analysis, Data curation, Conceptualization. **Li Han:** Writing – original draft, Project administration, Investigation, Formal analysis, Data curation, Conceptualization. **Alimu Aikebaier:** Project administration, Investigation, Formal analysis, Data curation. **Yanyan Shi:** Project administration, Investigation, Formal analysis, Data curation. **Gang Cheng:** Writing – review & editing, Validation, Supervision, Formal analysis, Conceptualization. **Lei Cheng:** Validation, Supervision, Formal analysis, Conceptualization. **Guo Ma:** Writing – review & editing, Validation, Supervision, Formal analysis, Conceptualization.

## Declaration of competing interest

All authors have no conflicts of interest relevant to this article to disclose.

## Appendix Supplementary materials

Image, image 1

Image, image 2

Image, image 3

Image, image 4

Image, image 5

Image, image 6

Image, image 7

Image, image 8

Image, image 9

Image, image 10

Image, image 11

Image, image 12

Image, image 13

Image, image 14

Image, image 15

Image, image 16

Image, image 17

Image, image 18

Image, image 19

Image, image 20

Image, image 21

Image, image 22

Image, image 23

Image, image 24

Image, image 25

Image, image 26

Image, image 27

Image, image 28

Image, image 29

Image, image 30

Image, application 31

Image, application 32
