# Supplementary material for: Cardiovascular Events and Heart Failure in Patients With Type 2 Diabetes Treated With Dipeptidyl Peptidase-4 Inhibitors: A Meta-Analysis
Source: Curr Ther Res Clin Exp. 2025 Jul 15;103:100804. doi: 10.1016/j.curtheres.2025.100804 (PMC12358673; doi:10.1016/j.curtheres.2025.100804)
Supplement: Supplementary file 31 [file mmc31.docx]

Supplemental Figures 1：Risk of bias graph

Supplemental Figures 1：Risk of bias summary

Supplemental Figures 2 : Funnel plot for MACE.

Supplemental Figures 3：subgroup analysis of MACE based on with or without cardiovascular endpoints trial

Supplemental Figures 3-1：subgroup analysis of MACE based on class of control drug

Supplemental Figures 3-2：subgroup analysis of MACE based on class of DPP-4
Supplemental Figures 3-3：subgroup analysis of MACE based on previous history of cardiovascular disease

Supplemental Figures 4：Funnel plot for all-cause mortality.

Supplemental Figures 5：subgroup analysis of all-cause mortality based on with or
without cardiovascular endpoints trial

Supplemental Figures 5-1 ：subgroup analysis of all-cause mortality based on
class of control drug
Supplemental Figures 5-2 ：subgroup analysis of all-cause mortality based on
class of DPP-4
Supplemental Figures 5-3: subgroup analysis of all-cause mortality based on previous history of
cardiovascular disease
Supplemental Figures 5-4：subgroup analysis of all-cause mortality based on class of follow-up weeks

Supplemental Figures 6：Funnel plot for HF.

Supplemental Figures 7: subgroup analysis of HF based on with or without cardiovascular endpoints trial

Supplemental Figures 7-1: subgroup analysis of HF based on class of control drug；Other: Su or TZD,
Su:sulfonylureas, TZD:Thiazolidinediones.
Supplemental Figures 7-2: subgroup analysis of HF based on class of DPP-4
Supplemental Figures 7-3: subgroup analysis of HF based on previous history of
cardiovascular disease
Supplemental Figures 7-4: subgroup analysis of HF based on class of follow-up weeks

Supplemental Figures 8: Funnel plot for MI.

Supplemental Figures 9: subgroup analysis of MI based on with or without cardiovascular endpoints trial

Supplemental Figures 9-1: subgroup analysis of MI based on class of control drug

Supplemental Figures 9-2: subgroup analysis of MI based on class of DPP-4
Supplemental Figures 9-3: subgroup analysis of MI based on class of follow-up weeks
Supplemental Figures 9-4: subgroup analysis of MI based on class of follow-up weeks
Supplemental Figures 10: Funnel plot for Strok.

Supplemental Figures 11: subgroup analysis of Strok based on with or without
cardiovascular endpoints trial

Supplemental Figures 11-1: subgroup analysis of Strok based on
class of control drug
Supplemental Figures 11-2: subgroup analysis of Strok based on
class of DPP-4
Supplemental Figures 11-3: subgroup analysis of Strok based on
previous history of cardiovascular disease
