# Supplementary material for: Cardiovascular Events and Heart Failure in Patients With Type 2 Diabetes Treated With Dipeptidyl Peptidase-4 Inhibitors: A Meta-Analysis
Source: Curr Ther Res Clin Exp. 2025 Jul 15;103:100804. doi: 10.1016/j.curtheres.2025.100804 (PMC12358673; doi:10.1016/j.curtheres.2025.100804)
Supplement: Supplementary file 32 [file mmc32.docx]

**Supplemental Tables 1:** Search strategy

| **Data source** | **Search terms** |
| --- | --- |
| **PubMed** | #1 Dipeptidyl-Peptidase IV Inhibitors  #2 Dipeptidyl Peptidase IV Inhibitors  #3 DPP-4 Inhibitor  #4 DPP 4 Inhibitor  #5 Inhibitor, DPP-4  #6 DPP-IV Inhibitor  #7 DPP IV Inhibitor  #8 Inhibitor, DPP-IV  #9 DPP-4 Inhibitors  #10 DPP 4 Inhibitors  #11 DPP-IV Inhibitors  #12 DPP IV Inhibitors  #13 Gliptin  #14 Dipeptidyl Peptidase 4 Inhibitor  #15 Dipeptidyl-Peptidase IV Inhibitor  #16 Dipeptidyl Peptidase IV Inhibitor  #17 Inhibitor, Dipeptidyl-Peptidase IV  #18 OR #1～ #17  #19 Omarigliptin  #20 Sitagliptin Phosphate OR Phosphate, Sitagliptin OR MK 0431 OR Sitagliptin Phosphate Anhydrous OR MK0431  #21 Omarigliptin OR NVP-LAF237 OR NVP LAF237 OR Galvus)  #22 saxagliptin OR Onglyza OR BMS 477118  #23 alogliptin OR benzonitrile OR nesina OR SYR 322 OR SYR-322  #24 trelagliptin OR SYR-472 OR trelagliptin succinate  #25 anagliptinOR anagliptin hydrochloride  #26 Linagliptin  #27 Gemigliptin  #28 OR #19～ #27  #29 random*  #30 "Randomized Controlled Trial  #31 RCT or RCTs  #32 OR #29~ #31  #33 #18 AND #28 AND #32 |
| **Embase** | Title-Abstract-Author Keywords ((‘Dipeptidyl-Peptidase IV Inhibitors’ OR ‘Dipeptidyl Peptidase IV Inhibitors’ OR ‘DPP-4 Inhibitor’ OR ‘Inhibitor, DPP-4’ OR ‘Vildagliptin’ OR ‘Sitagliptin Phosphate’ OR ‘Omarigliptin’ OR ‘saxagliptin’ OR ‘alogliptin’ OR ‘trelagliptin’ OR ‘anagliptin’ OR ‘Linagliptin’ OR ‘Gemigliptin’ OR ‘evogliptin’) AND TITLE-ABSTRACT-INDEX TERM (RCT* OR random*) |
| **Cochrane** | #1(Diabetes Mellitus): ti,ab,kw  #2(Dipeptidyl-Peptidase IV Inhibitors or Dipeptidyl Peptidase IV Inhibitors or S DPP-4 Inhibitor or DPP 4 Inhibitor or Inhibitor, DPP-4 or DPP-IV Inhibitor or DPP IV Inhibitor or Inhibitor, DPP-IV or DPP-4 Inhibitors or DPP 4 Inhibitors or DPP-IV Inhibitors or Gliptin or Dipeptidyl Peptidase 4 Inhibitor or Inhibitor, Dipeptidyl-Peptidase IV):ti,ab,kw  #3(Vildagliptin or NVP-LAF237 or NVP LAF237 or Galvus):ti,ab,kw  #4(Sitagliptin Phosphate or Phosphate, Sitagliptin or Sitagliptin Phosphate Monohydrate or MK 0431, MK-0431):ti,ab,kw  #5(Omarigliptin or MK-3102):ti,ab,kw  #6(saxagliptin or Onglyza or BMS 477118 or BMS477118 or BMS-477118):ti,ab,kw  #7(alogliptin or benzonitrile or nesina or SYR 322):ti,ab,kw  #8(trelagliptin or SYR-472 or trelagliptin succinate):ti,ab,kw  #9(anagliptin or anagliptin hydrochloride):ti,ab,kw  #10(Gemigliptin or LC15-0444):ti,ab,kw  #11(evogliptin):ti,ab,kw  #12(teneligliptin):ti,ab,kw  OR #2～#12  #13(randomized controlled trial OR randomized controlled trials OR randomised controlled trial OR randomized OR clinical trials, randomized OR trials, randomized clinical OR controlled clinical trials, randomized):ti,ab,kw  AND#1～#13 |
| **ClinicalTrials.gov** | (teneligliptin OR evogliptin OR Gemigliptin OR Linagliptin OR anagliptin OR trelagliptin OR alogliptin OR saxagliptin OR Omarigliptin OR Sitagliptin Phosphate) |
|  |  |

**Supplemental Tables 2** – Principal characteristics of trials included in the metanalysis.

| **Author** | **Year** | **NTC Number** | **Intervention** | **Control** | **Trial duration(weeks)** | **Patients(n)** | **Age (years)** | **Female (%)** | **BMI (Kg/m^2^)** | **HbA1c  (%)** | **Diabeta duration (years)** |
| --- | --- | --- | --- | --- | --- | --- | --- | --- | --- | --- | --- |
| Rosenstock | 2019 | NCT01243424 | Linagliptin | Glimepiride | 302 | 6033 | 64 | 40.01 | 30.1 | 7.2 | 6.3 |
| Ahren | 2014 | NCT00838903 | Sitagliptin | Placebo | 156 | 1012 | 54.5 | 52.4 | NR | 8.1 | 6.3 |
|  |  |  |  | Glimepiride |  |  |  |  |  |  |  |
|  |  |  |  | Albiglutide |  |  |  |  |  |  |  |
| Ferreira | 2013 | NCT00509236 | Sitagliptin | Glipizide | 54 | 129 | 59.5 | 52 | 26.8 | 7.6 | 17.5 |
| Barnett | 2013 | NCT00757588 | Saxagliptin | Placebo | 52 | 455 | 57.2 | 58.7 | 32.2 | 8.7 | 12 |
| Chen | 2018 | NCT02104804 | Saxagliptin | Placebo | 24 | 462 | 59.1 | 54.8 | NR | 8.53 | 13.4 |
| Ahren | 2017 | NCT01930188 | Sitagliptin | Semaglutide | 56 | 613 | 55.1 | 49.3 | 32.5 | 8.1 | 6.6 |
| Chacra | 2017 | NCT01698775 | omarigliptin | Placebo | 24 | 213 | 65.2 | 38.5 | 30.1 | 8.3 | 15 |
| Pollock | 2019 | NCT02547935 | Saxagliptin | Dapagliflozin | 24 | 448 | 64.5 | 70.7 | 30.4 | 8.4 | NR |
|  |  |  |  | Placebo |  |  |  |  |  |  |  |
| Rosenstock | 2019 | NCT02607865 | Sitagliptin | Semaglutide | 78 | 933 | 58 | 47.3 | 32.6 | 8.3 | 8.6 |
| Halvorsen | 2019 | NCT03115112 | Sitagliptin | Bexagliflozin | 24 | 384 | 59.4 | 35.9 | 31.7 | 8 | 8.79 |
| Ledesma | 2019 | NCT02240680 | linagliptin | Placebo | 52 | 302 | 72.4 | 39.4 | 28.09 | 8.2 | NR |
| Matthews | 2019 | NCT01528254 | Vildagliptin | Placebo | 302 | 2001 | 54.3 | 53 | 31.1 | 6.7 | 6.4 |
| Pieber | 2019 | NCT02849080 | Sitagliptin | Semaglutide | 52 | 504 | 57.4 | 43.5 | 31.5 | 8.3 | 8.8 |
| White | 2018 | NCT00968708 | Alogliptin | Placebo | 160 | 1398 | 59.9 | 70.3 | 29 | 8.1 | 8.7 |
| Umpierrez | 2017 | NCT02061969 | Linagliptin | Glargine | 24 | 140 | 69.8 | 59 | 29.9 | 7.88 | 10.7 |
| Green | 2015 | NCT00790205 | Sitagliptin | Placebo | 272 | 14671 | 65.5 | 29.3 | 30.2 | 7.2 | 11.6 |
| Dobs | 2013 | NCT00350779 | Sitagliptin | Placebo | 54 | 262 | 54.6 | 42 | 30.5 | 8.8 | 9.6 |
| Gantz | 2017 | NCT01703208 | Omarigliptin | Placebo | 234 | 4202 | 63.6 | 30.9 | 31.3 | 8.1 | 12.1 |
| Schernthaner | 2015 | NCT01006603 | Saxagliptin | Glimepiride | 52 | 720 | 72.6 | 38.2 | 29.6 | 7.6 | 7.6 |
| Hartley | 2015 | NCT01189890 | Sitagliptin | Glimepiride | 32 | 388 | 70.7 | 56.3 | 29.7 | 7.8 | 8.7 |
| Laakso | 2015 | NCT01087502 | Linagliptin | Glimepiride | 52 | 235 | 66.2 | 36.6 | 32.09 | 8.05 | NR |
| Rosenstock | 2013 | NCT00707993 | Alogliptin | Glipizide | 52 | 441 | 69.9 | 55.1 | 29.8 | 7.5 | 6.1 |
| Mosenzon | 2015 | NCT01107886 | Saxagliptin | Placebo | 101 | 16492 | 65.1 | 33.1 | 30.6 | 7.5 | NR |
| Yki-Järvinen | 2013 | NCT00954447 | linagliptin | Placebo | 52 | 1261 | 60.1 | 48.2 | 31 | 8.3 | NR |
| Göke | 2013 | NCT00575588 | Saxagliptin | Glipizide | 52 | 858 | 57.5 | 48.3 | 31.4 | 7.7 | 5.5 |
| Nowicki | 2011 | NCT00614939 | Saxagliptin | Placebo | 52 | 170 | 67 | 57.1 | 30.7 | 8.3 | NR |
| Pfützner | 2011 | NCT00327015 | Saxagliptin | Metformin | 76 | 663 | 52.1 | 50.8 | 30.2 | 9.5 | 1.7 |
| Hollander | 2011 | NCT00295633 | Saxagliptin | Thiazolidinedione | 24 | 379 | 54.4 | 50.4 | 30.2 | 8.3 | 5.2 |
| Pratley | 2009 | NCT00286494 | Alogliptin | pioglitazone | 26 | 296 | 55.4 | 41.8 | 32.8 | 8 | 7.6 |
| Pratley | 2010 | NCT00700817 | sitagliptin | liraglutide | 78 | 440 | 55 | 46.5 | 32.9 | 8.4 | 6.4 |
| Gallwitz | 2012 | NCT00622284 | Linagliptin | Glimepiride | 96 | 1551 | 59.8 | 39.5 | 30.2 | 7.7 | NR |
| Nauck | 2009 | NCT00286442 | Alogliptin | Placebo | 26 | 311 | 55 | 49.7 | 32 | 8 | 6 |
| Nauck | 2007 | NCT00094770 | Sitagliptin | Glipizide | 52 | 1172 | 56.7 | 40.8 | 31.2 | 7.7 | 6.4 |
| Cooper | 2020 | NCT01897532 | Linagliptin | Placebo | 206 | 6979 | 65.9 | 37.1 | 31.1 | 7.9 | 14.4 |
| Groop | 2017 | NCT01792518 | Linagliptin | Placebo | 24 | 360 | 60.6 | 36.4 | 28.5 | 7.85 | 10.2 |
| Softeland | 2016 | NCT01734785 | Linagliptin | Empagliflozin | 24 | 327 | 55.2 | 40.4 | 30.6 | 8 | NR |
| Sugawara | 2023 | jRCTs031180241 | DPP4 | Luseogliflozin | 52 | 549 | 57.8 | 34.7 | NR | 7.7 | 4.5 |
| Nagao | 2023 | UMIN000010376 | Sitagliptin | Placebo | 24 | 176 | 71.3 | 42 | 24.8 | 7.7 | NR |
| Webb | 2020 | NCT02043054 | Sitagliptin | liraglutide | 26 | 76 | 44.6 | 54.4 | 35.6 | 7.5 | 4.5 |
| Wexler | 2023 | NCT01794143 | Sitagliptin | Glimepiride | 240 | 5047 | 57.2 | 36.4 | 34.3 | 7.5 | 4.2 |
|  |  |  |  | liraglutide |  |  |  |  |  |  |  |
|  |  |  |  | glargine |  |  |  |  |  |  |  |
| Lewin | 2015 | NCT01422876 | Linagliptin | Empagliflozin | 52 | 552 | 54.6 | 46.2 | 31.6 | 8 | NR |
| Rosenstock | 2015 | NCT01606007 | Saxagliptin | Dapagliflozin | 24 | 534 | 54 | 50 | 31.7 | 8.9 | 7.6 |
| Lavalle-Gonzalez | 2013 | NCT01106677 | Sitagliptin | Canagglifiozin | 52 | 1284 | 55.4 | 52.9 | 31.8 | 7.9 | 6.9 |
| Schernthaner | 2013 | NCT01137812 | Sitagliptin | Canagglifiozin | 52 | 755 | 56 | 44.1 | 31.6 | 8.1 | 9.6 |
| Scott | 2018 | NCT02532855 | Sitagliptin | Canagglifiozin | 26 | 584 | 55 | 55.8 | 31.6 | 8 | 4.3 |
| Stenlof | 2014 | NCT01081834 | Sitagliptin | Dapagliflozin | 24 | 314 | 67.1 | 42.1 | 31.6 | 7.7 | 10.6 |
| Ferrannini | 2013 | NCT00881530 | Sitagliptin | Empagliflozin | 78 | 388 | 60 | 48.2 | 27 | 7.9 | NR |
| Roden | 2013 | NCT01177813 | Sitagliptin | Empagliflozin | 24 | 899 | 55 | 39 | 284 | 7.9 | NR |
| Roden | 2015 | NCT01289990 | Sitagliptin | Empagliflozin | 76 | 647 | 55 | 38.7 | 28.4 | 7.88 | NR |

NR: Not Report
